# Microbiologically Influenced Corrosion of Carbon Steel Beneath a Deposit in CO_2_-Saturated Formation Water Containing *Desulfotomaculum nigrificans*

**DOI:** 10.3389/fmicb.2019.01298

**Published:** 2019-06-12

**Authors:** Hongwei Liu, Guozhuo Meng, Weihua Li, Tingyue Gu, Hongfang Liu

**Affiliations:** ^1^School of Chemical Engineering and Technology, Sun Yat-sen University, Zhuhai, China; ^2^Department of Chemical and Biomolecular Engineering, Institute for Corrosion and Multiphase Technology, Ohio University, Athens, OH, United States; ^3^Key Laboratory of Material Chemistry for Energy Conversion and Storage, Ministry of Education, Hubei Key Laboratory of Materials Chemistry and Service Failure, School of Chemistry and Chemical Engineering, Huazhong University of Science & Technology, Wuhan, China

**Keywords:** sulfate reducing bacteria, biofilm, carbon steel, under deposit corrosion, microbiological corrosion

## Abstract

The corrosion mechanism of carbon steel under deposit in the presence of sulfate reducing bacterium (SRB) *Desulfotomaculum nigrificans* was studied using surface analysis, weight loss and electrochemical measurements. Results showed that both the general corrosion and localized corrosion were considerably promoted by SRB under deposit. The corrosion rate of steel in the presence of SRB was approximately 6 times of that for the control according to the weight loss measurements. The maximum corrosion pit depth in the presence of SRB was approximately 7.7 times of that of the control. Both the anodic and cathodic reactions were significantly accelerated by SRB. A galvanic effect in the presence of SRB due to the heterogeneous biofilm led to serious localized corrosion.

## Introduction

In the oil and gas fields, pipeline steel corrosion not only resulted in large economic losses but caused safety related accidents (da Costa Mattos et al., 2016; Feng and Cheng, 2017). Large amounts of solid particles can accumulate in the interior of a pipeline, which can bring under deposit corrosion (UDC) (Alanazi et al., 2014; Zhu et al., 2015). UDC is a key reason for serious localized corrosion. Some researchers have investigated UDC under different testing conditions and with different deposits. Han et al. (2013) studied steel corrosion under sand deposit, and their results showed that corrosion pits were initiated by a galvanic effect. Standlee et al. (2011) found that FeS deposit could considerably accelerate steel corrosion compared with those in the sand deposit, and serious pitting corrosion with mill scale was also observed. Hoseinieh et al. (2016) investigated steel corrosion covered by calcareous deposit using electrochemical noise, and found that there were two different stages for localized corrosion processes. The UDC is chemically and physically different from the bare steel corrosion in the absence of deposit, and the concentration of aggressive species and pH also differ (Huang et al., 2010).

There are only a few reports that combined microbiologically influenced corrosion (MIC) and UDC. Rahmani et al. (Pandarinathan et al., 2013a) found that SRB promoted localized corrosion under calcium carbonate deposit. Sulfate reducing bacteria (SRB) are commonly found in the oil and gas fields, which are the main anaerobic corrosive microorganisms causing MIC (Guan et al., 2013; Li X.-X. et al., 2016; Voordouw et al., 2016). There are no reports about SRB corrosion under deposit in the sour oil and gas fields. SRB use sulfate as the terminal electron acceptor, and can considerably accelerate steel corrosion, especially for localized corrosion (Enning and Garrelfs, 2014). The formation of SRB biofilm on steel surface can affect the kinetics of anodic and cathodic reactions, leading to an acceleration of steel corrosion (Beech and Sunner, 2004; Zuo, 2007). Extracellular polymeric substances (EPS) secreted by microorganisms also play a role in steel corrosion (Dong et al., 2011a; Liu et al., 2017a). SRB can directly switch electrons from elemental iron via extracellular electron transfer, then cause pitting attacks (Li et al., 2018; Gu et al., 2019; Jia et al., 2019). SRB are anaerobic microbes. If there is oxygen in the environment, SRB can grow beneath another biofilm that consumes oxygen to provide a locally anaerobic environment. Thus, SRB can grow well beneath deposit. In sour oil and gas fields, CO_2_ corrosion is common. Under-deposit CO_2_ corrosion occurs, and it is different from the typical CO_2_ corrosion (Huang et al., 2010). Steel corrosion due to UDC is exacerbated by CO_2_ corrosion (Pandarinathan et al., 2013a). SRB MIC and CO_2_ corrosion can have a synergistic effect accelerating steel corrosion (Liu et al., 2017b). Study of SRB corrosion under deposit in the presence of CO_2_ corrosion is very meaningful.

Wire beam electrode (WBE), also known as array electrode, has been verified as a useful tool to study UDC (Tan et al., 2011; Hoseinieh et al., 2016) and MIC (Dong et al., 2011b). WBE is suitable for studying localized corrosion. The anodic and cathodic locations and their area ratio can be identified from the distributions of potential and current.

In sour oil and gas fields, there are solid particles in the interior of pipelines, which are derived from the sand and clay used in the exploration process (Hinds and Turnbull, 2010; Zhang et al., 2014). Thus, sand and clay were chosen as the materials for deposit in this paper.

In this paper, SRB corrosion of deposit-covered Q235 carbon steel in CO_2_-saturated formation water was studied using electrochemical measurements and surface characterization. WBE was used to investigate the initiation and propagation of localized corrosion. This paper aimed to get a better understanding of localized UDC of Q235 carbon steel in the presence of SRB and CO_2_.

## Experimental

### Specimen Preparation

The Q235 carbon steel used in this paper has a chemical composition (wt.%) of C 0.3, Si 0.01, Mn 0.42, S 0.029, P 0.01, and Fe balance. Specimens with an exposed surface area of 0.785 cm^2^ and specimens with dimensions of 50 × 13 × 1.5 mm were used for electrochemical and weight loss measurements, respectively. Specimens with a diameter of 15 mm and thickness of 3 mm were used for biofilm observation and EDS analysis. Prior to testing, all specimens were abraded through 600, 800, and 1200-grit silicon carbide papers, then degreased in acetone and washed with anhydrous ethanol. All specimens were sanitized under a UV lamp for 30 min before use.

### Water Sample

A water sample was prepared according to the composition of the formation water produced from an oil field (Zhang et al., 2014). Table 1 shows the chemical composition of the artificial formation water used in this work.

**Table 1:** Chemical composition of artificial formation water (g L^−1^).

KCl	CaCl_2_	Na_2_SO_4_	NaHCO_3_	NaCl	MgCl_2_ ∙ 6H_2_O
0.54	0.45	0.37	3.98	17.24	0.5

### Preparation of Deposit

The mixture of sand and clay with a dry mass ratio of 5:1 was used as deposit. The sand particle sizes were in the range of 0.2∼0.4 mm. The sand particles were immersed in boiling water for 1 h, then rinsed with sulfuric acid, acetone and deionized water sequentially. The clay was commercially purchased from chemical Ltd. The purity of clay is more than 99.5%. Prior to use, the dry deposit was sanitized under a UV lamp for 30 min. The thickness of the deposit covered on the steel surface was 3 mm during testing. A cylindrical ring of 3 mm was made to control the deposit thickness.

### Microbe Inoculation and Cultivation

*Desulfotomaculum nigrificans* identified in a previous study was used in this work (Liu et al., 2015b). The culture medium for the SRB seed culture had a composition of (g L^−1^): MgSO_4_ ⋅ 7H_2_O 0.2, K_2_HPO_4_ 0.01, NaCl 10, yeast extract 1.0, (NH)_2_Fe(SO_4_)_2_ 0.2, vitamin C 0.1, in addition to 4.0 mL L^−1^ sodium lactate. The culture medium had an initial pH of 7.2. The simulated formation water was seeded with 10% (v/v) of the SRB seed culture and then incubated at 37°C. Before inoculation, the simulated formation water was autoclaved at 121°C for 20 min. After that, it was deaerated by sparging CO_2_ gas (purity 99.95% by volume) for 4 h. The pH value of the simulated formation water was 6.15. The planktonic and sessile SRB cell counts were measured using the most probable number (MPN) method with an MPN culture medium (Liu et al., 2015b). The simulated formation water seeded with 10% (v/v) of the sterilized SRB culture medium was used as the control testing solution.

### Weight Loss Measurement

All specimens were taken out after 14 days of incubation. Deionized water and a pickling solution containing a corrosion inhibitor (imidazoline derivative) were used to remove deposits and corrosion products, respectively. Finally, all exposed specimen surfaces were rinsed with deionized water, cleaned with absolute ethanol, and dried under N_2_. Steel corrosion rates were assessed from the specific weight loss based on the exposed surface area.

### Characterization of Biofilm, Corrosion Surface Morphology, and Corrosion Products

Before scanning electron microscopy (SEM) and energy dispersive x-ray spectrum (EDS) analyses, the specimens for biofilm analysis were taken out after 14 days of incubation, using deionized water to remove deposit. Then the specimens were immersed in PBS solution containing 2.5% (w/w) glutaraldehyde for 8 h to immobilize sessile SRB cells (Liu and Cheng, 2018). After that, the specimens were dehydrated using ethanol with different concentrations in series and finally dried using N_2_. A thin gold film was coated on to the biofilm surface to provide conductivity.

A three-dimensional stereoscopic microscope (Model VHX-10000, Keyence, Japan) was used to observe the pit morphology after removing corrosion products. The corrosion products were identified using X-ray diffraction (XRD). XRD patterns were recorded with a diffractometer with Cu K_α_ radiation (Model PANalytical X’pert PRODY2198, Holland).

### Electrochemical Measurements

**FIGURE 1:**
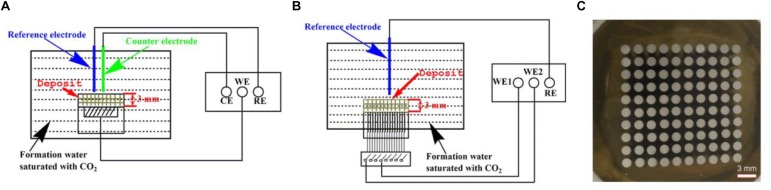
Schematic diagrams of the setup for electrochemical measurements (OCP, EIS, and potentiodynamic polarization curves) **(A)**, the setup for potential and galvanic current measurements **(B)** using a WBE **(C)**.

Open circuit potential (OCP), electrochemical impedance spectroscopy (EIS), and potentiodynamic polarization curves were conducted using an electrochemical workstation (Model CS350, Corrtest, China). The setup for the electrochemical measurements is illustrated in Figure 1A. The reference electrode and the counter electrode were saturated calomel electrode (SCE) and a platinum plate, respectively. EIS measurements begun after a steady-state OCP value was achieved by applying a sinusoidal voltage signal of 10 mV in a frequency range of 10^−2^ to 10^5^ Hz.

Potentiodynamic polarization curves were obtained at a sweep rate of 0.5 mV s^−1^ after EIS measurements with a scanning potential range of −250 to +350 mV versus OCP. EIS data and polarization curves were analyzed using Zview2 software (Scribner, Inc.) and Cview2 software (Scribner, Inc.).

### Preparation of Wire Beam Electrode and Potential and Current Scans

The WBE was composed of 100 pieces of Q235 carbon steel disks with a diameter of 1.5 mm as shown in Figure 1C. The potential and current scans were conducted using an electrochemical instrument (Model CST520, Corrtest, China). A SCE was used as the reference electrode. A 10 × 10 autoswitch array was used to switch among the individual electrodes on the WBE shown in Figure 1B, so each tiny electrode was used as the working electrode one at a time. All 100 electrodes were connected to each other when no measurements were performed.

## Results

### SRB Cell Counts

Table 2 shows SRB cell counts after 14 days of incubation. It is seen that both planktonic and sessile SRB could survive well, thus causing MIC. The sessile SRB cells under deposit, i.e., the sessile SRB in biofilm, increased one order of magnitude compared with the planktonic SRB. The sessile SRB are closely related to MIC. The higher sessile SRB cells mean the more serious steel corrosion.

**Table 2:** SRB cell counts after 14 days of incubation.

0 day (planktonic, cell mL^−1^)	14 days (planktonic, cell mL^−1^)	14 days (sessile, cell cm^−2^)
(2.8 ± 0.6) × 10^5^	(6.2 ± 1.9) × 10^5^	(5.1 ± 1.3) × 10^6^

*Standard deviations from three independent samples.*

### SEM Analysis of SRB Biofilms

**FIGURE 2:**
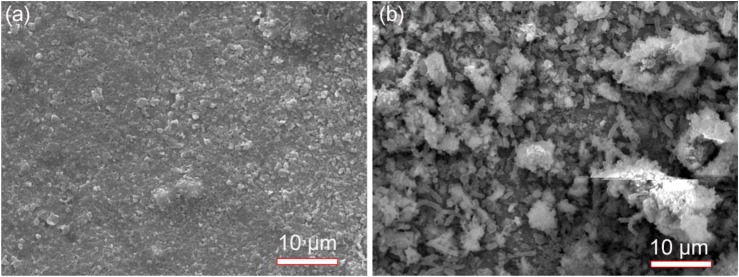
Scanning electron microscopy images of specimens with covered deposit after 14-days of incubation in the absence **(a)** and presence **(b)** of SRB in CO_2_-saturated simulated formation water.

Scanning electron microscopy images of specimens in the absence and presence of SRB after 14 days of incubation are shown in Figure 2. In this paper, the specimen covered with deposit in the absence of SRB is the control. The corrosion product film in Figure 2a appeared thin and dense in the absence of SRB. A dense corrosion product film usually had a good inhibition effect against further corrosion. The SEM image in the presence of SRB, shown in Figure 2b, loose quite different. Numerous sessile SRB cells embedded in corrosion products. The corrosion product film appeared thicker but porous. This porous film allowed mass transfer of nutrients to SRB cells inside the film. It did not offer corrosion inhibition.

**FIGURE 3:**
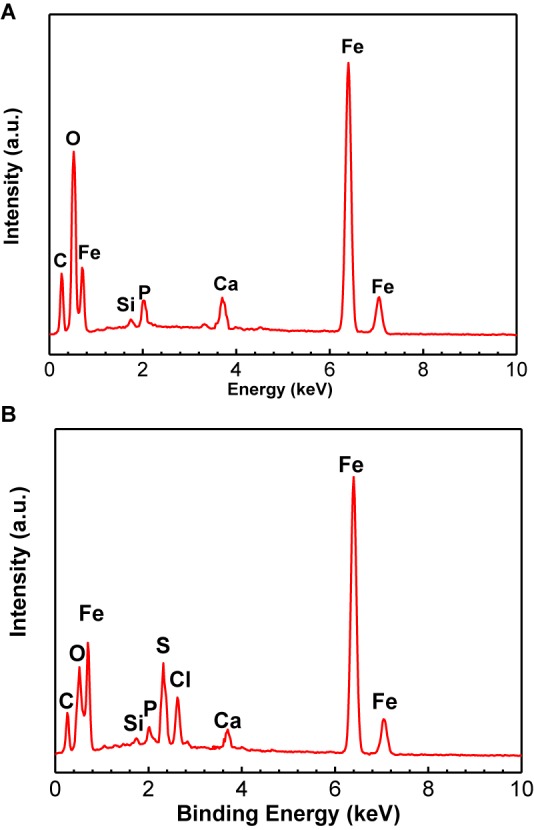
Energy dispersive x-ray spectrum analysis of corrosion product film and SRB biofilm on specimens after 14-days of incubation in the absence **(A)** and presence **(B)** of SRB, respectively.

The EDS analysis results are shown in the Figure 3 and Table 3. The Fe, P, Ca, Si, and O elements could be found for the control specimen. The higher content of S element (8.16 wt%) was found in the presence of SRB, which indirectly verified the presence of SRB corrosion. FeS is the typical corrosion products of SRB. The presence of S element further demonstrates that SRB can grow well under deposit. A little amount of Cl^−^ in the presence of SRB is found but is absent for the control specimen, which can be due to the structure difference of corrosion product films.

**Table 3:** Energy dispersive x-ray spectrum analysis results of corrosion product film and SRB biofilm on specimens after 14-days of incubation in the absence and presence of SRB, respectively.

	C	O	Fe	Si	Ca	P	S
Control	13.42	18.30	60.63	0.28	4.81	2.56	–
SRB	10.81	9.15	69.86	0.36	1.17	0.49	8.16

### Weight Loss

**FIGURE 4:**
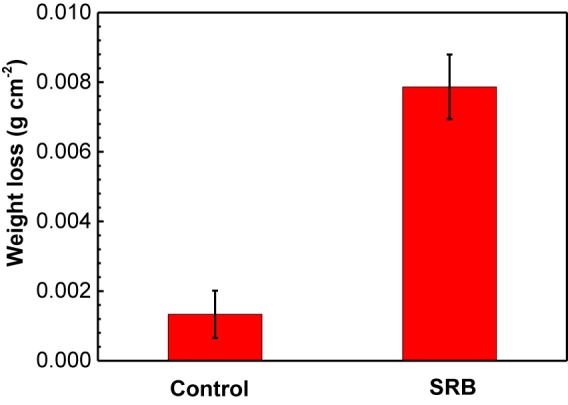
Weight loss results of specimens covered with deposit in the absence and presence of SRB after 14-days of incubation in CO_2_-saturated simulated formation water. (Error bars represent standard deviation).

Figure 4 shows the weight loss measurement results of specimens under deposit in the absence and presence of SRB after 14 days of incubation. For the control specimen, it is seen that corrosion was very limited with a lower weight loss (1.3 ± 0.7 mg cm^−2^). The weight loss increased by approximately 6 times in the presence of SRB, with a higher weight loss (7.9 ± 0.9 mg cm^−2^). These results indicated that SRB considerably accelerated UDC corrosion.

### XRD Analysis

**FIGURE 5:**
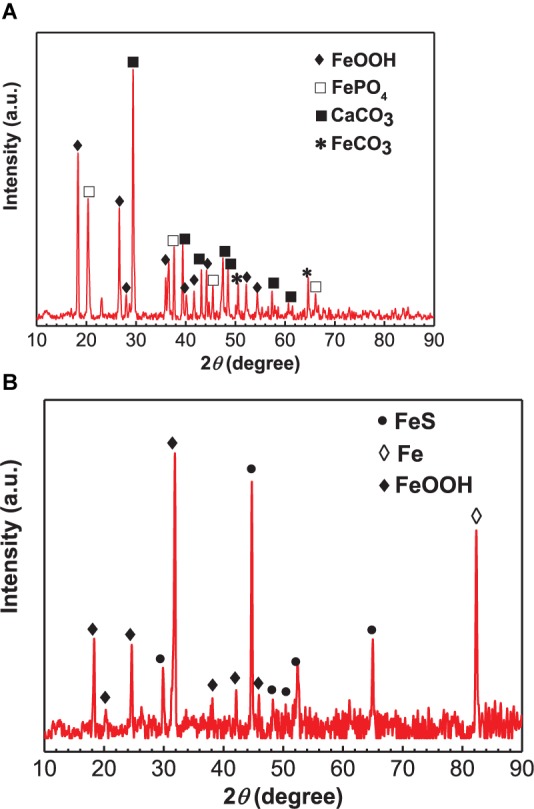
X-ray diffraction analysis of corrosion products on specimens covered with deposit in the absence **(A)** and presence of SRB **(B)** after 14 days of incubation in CO_2_-saturated simulated formation water.

The XRD analysis results of corrosion products in the absence and presence of SRB are shown in Figure 5. For the control specimen, the corrosion products were composed of CaCO_3_, FePO_4_, FeOOH, and little FeCO_3_ (Figure 5A). The formation of a CaCO_3_ scale apparently offered corrosion inhibition and led to a light corrosion. In the presence of SRB, the corrosion products included FeS (PDF Card 370477), and FeOOH (Figure 5B). FeS is the typical corrosion product of SRB MIC. At a pH above 6.5, some Fe^2+^ was precipitated as Fe(OH)_2_ (Liu et al., 2015b). A small amount of Fe(OH)_2_ was oxidized to FeOOH by O_2_ during the specimen preparation process for XRD analysis rather than a corrosion product during incubation (Liu et al., 2016a).

### Surface Morphology After Removing Corrosion Products

**FIGURE 6:**
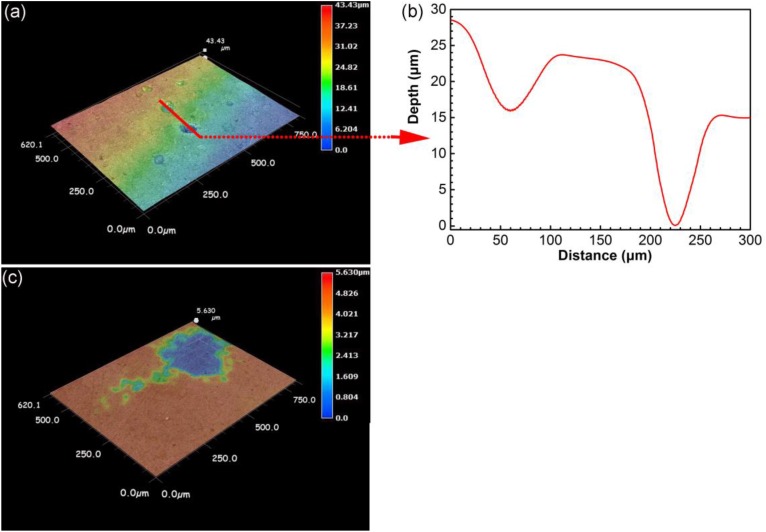
Surface morphology after removing corrosion products for specimens after 14 days of incubation in the presence **(a,b)** and in the absence **(c)** of SRB in CO_2_-saturated simulated formation water.

Figure 6 shows the surface morphology of specimens after removing corrosion products after 14 days of incubation. Serious pitting corrosion could be observed on the surface of the specimen incubated in the presence of SRB with the maximum pit depth of 43.4 μm (Figure 6a), which was approximately 7.7 times of that on the control specimen (5.6 μm). This proved that SRB accelerated UDC with CO_2_.

### Electrochemical Measurements

#### OCP

**FIGURE 7:**
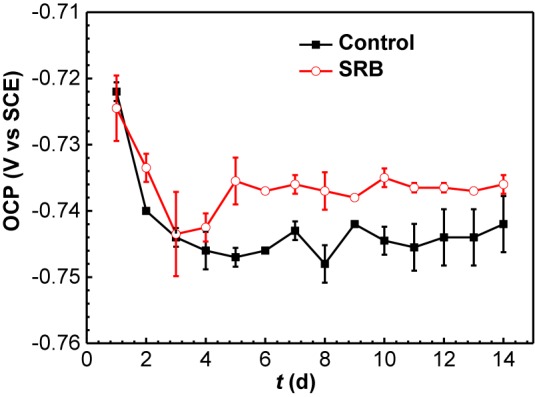
Changes of OCP values with time in the absence and presence of SRB in CO_2_-saturated simulated formation water. (Error bars represent standard deviations).

Figure 7 shows the changes of OCP with time in the absence (control) and presence of SRB. For the control specimen, OCP declined during the initial 4 days, then became relatively stable. In the presence of SRB, OCP declined in the initial 3 days, then it increased for 2 days. After that, the OCP remained steady. The increase of OCP could be attributed to the formation of the SRB biofilm, which made its OCP consistently higher about that for the control after 3 days.

#### EIS Analysis

**FIGURE 8:**
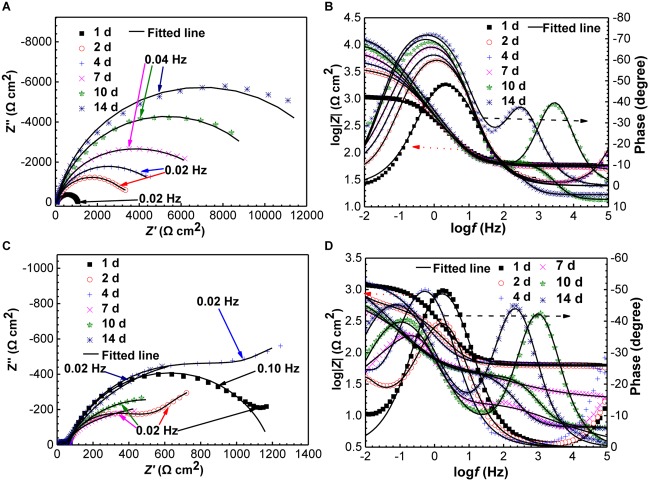
Changes of Nyquist and Bode plots of specimens with time in the absence **(A,B)** and presence **(C,D)** of SRB in CO_2_-saturated formation water.

The Nyquist and Bode plots of specimens in the absence and presence of SRB are shown in Figure 8. The diameter of the Nyquist plots for the control specimen at the 1 day was very smaller, indicating UDC (Figure 8A). Then, the diameters increased gradually with time (Figure 8A). A two-time constant behavior can be clearly observed in the bode plots in Figure 8B after 10 days of incubation, which corresponded to the formation of a dense corrosion product film. The dense film increased the impedance. In the presence of SRB, the diameters of Nyquist plots were obviously smaller than those for the control (Figure 8C), which further indicates that SRB accelerated UDC. The diameters of Nyquist plots decreased initially in the first 2 days, then increased until 7 days (Figure 8C). Finally, the diameters of the Nyquist plots changed much less with time. The presence of Warburg impedances could be attributed to the formation of a SRB corrosion product film, resulting in diffusion control. But the Warburg impedances disappeared due to the change of biofilm structure with incubation time. From the Bode plots in Figure 8D, a two-time constant behavior can be found after 7 days of incubation, which also pointed to the formation of SRB biofilm, which was responsible for MIC.

**FIGURE 9:**
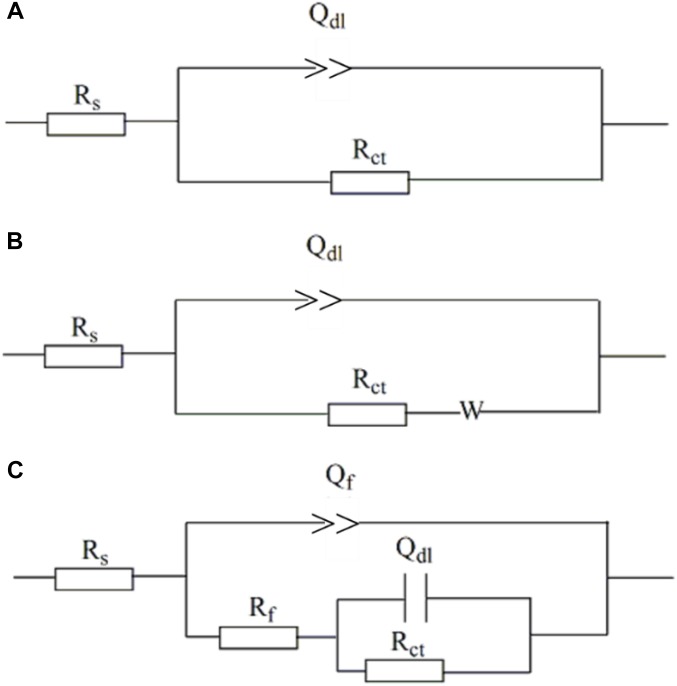
Electro chemical equivalent circuits used to fit the EIS data. **(A)** One-time constant; **(B)** one-time constant with Warburg impedance; **(C)** one-time constant.

Figure 9 shows the equivalent circuits used to fit EIS data. All the EIS data were fitted well (Figure 8) with a smaller fitted error (<10%), especially with the use of the constant-phase element (Q) to replace capacitance due to the heterogeneity of specimens. The impedance of Q, i.e., Z_Q_, was calculated using the equation below,

(1) $$Z_{\text{Q}}=Y_{0}{}^{-1}{\left(j\omega \right)}^{-\alpha }$$

where ω is angular frequency (rad s^−1^), and *Y*_0_ and α are exponents indicating the deviation of the specimen from the ideal capacitive behavior (Chongdar et al., 2005). In the equivalent circuits in Figure 8, *R*_s_ is solution resistance, *R*_f_ and *Q*_f_ the resistance and constant-phase element of corrosion product film, respectively. *R*_ct_ and *Q*_dl_ are charge-transfer resistance and constant-phase element of the double-charge layer, respectively, and *W* the Warburg impedance.

**FIGURE 10:**
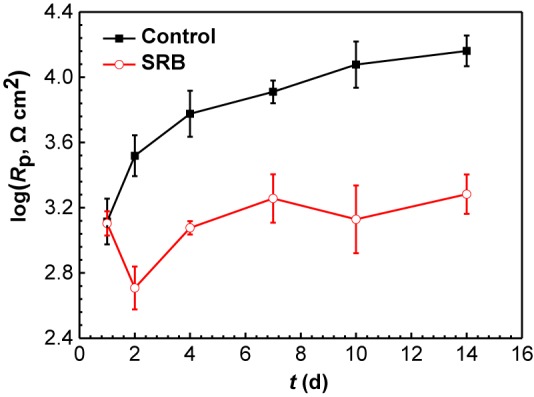
Changes of *R*_p_ values from EIS data in the Figure 8 in the absence and presence of SRB. (Error bars represent standard deviations).

Figure 10 shows the changes of *R*_p_ values fitted EIS data, and *R*_p_ values are the summation of *R*_f_ and *R*_ct_ values. *R*_p_ is inversely proportional to the corrosion rate, meaning a higher *R*_p_ value corresponds to a lower corrosion rate (Liu et al., 2016b). It is seen that *R*_p_ increased gradually with time for the control specimen, while in the presence of SRB, it dipped initially and after 2 days it until day 7 climbed. The *R*_p_ values in the presence of SRB were much smaller than that of the control, which meant higher corrosion with SRB. The EIS results corrugated to weight loss results (Figure 4).

#### Potentiodynamic Polarization Curve Analysis

**FIGURE 11:**
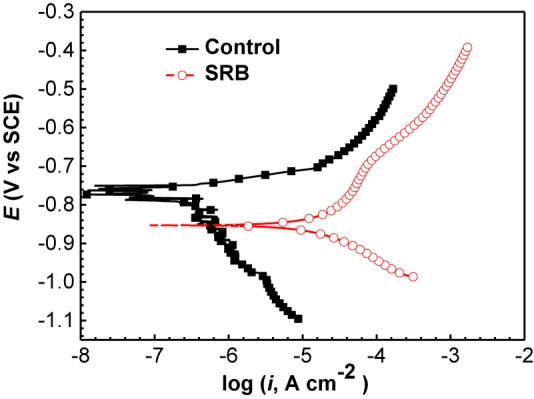
Potentiodynamic polarization curves of specimens covered with deposit in the absence and presence of SRB after 14 days of incubation in CO_2_-saturated simulated formation water.

Figure 11 shows the potentiodynamic polarization curves of the specimens incubated in the absence and presence of SRB for 14 days. The corresponding electrochemical parameters are shown in Table 4. It is seen that the corrosion current density (*i*_corr_) with a value of (1.71 ± 0.86) × 10^−5^ A cm^−2^ in the presence of SRB was considerably larger than the *i*_corr_ value of (1.32 ± 0.46) × 10^−6^ A cm^−2^ for the control (Table 4). The value of *i*_corr_ is directly proportional to the steel corrosion rate (Liu et al., 2015b). Thus, the analysis here suggests that SRB accelerated UDC. In Figure 11, it is seen that both the anodic and cathodic reactions were accelerated in the presence of SRB. This was especially true for the cathodic reaction. The difference of E_corr_ with OCP can be due to the change of surface films in the absence and presence of SRB.

**Table 4:** Electrochemical parameters fitted from the potentiodynamic polarization data in the absence and presence of SRB after 14 days of incubation.

	*b*_a_ (V dec^−1^)	*b*_c_ (V dec^−1^)	*E*_corr_ (V vs. SCE)	*i*_corr_ (A cm^−2^)
Control	0.131 ± 0.015	−0.349 ± 0.039	−0.765 ± 0.012	(1.32 ± 0.46) × 10^−6^
SRB	0.372 ± 0.053	−0.102 ± 0.024	−0.881 ± 0.019	(1.71 ± 0.86) × 10^−5^

*Errors represent standard deviations.*

#### Potential and Galvanic Current Distributions of WBE

**FIGURE 12:**
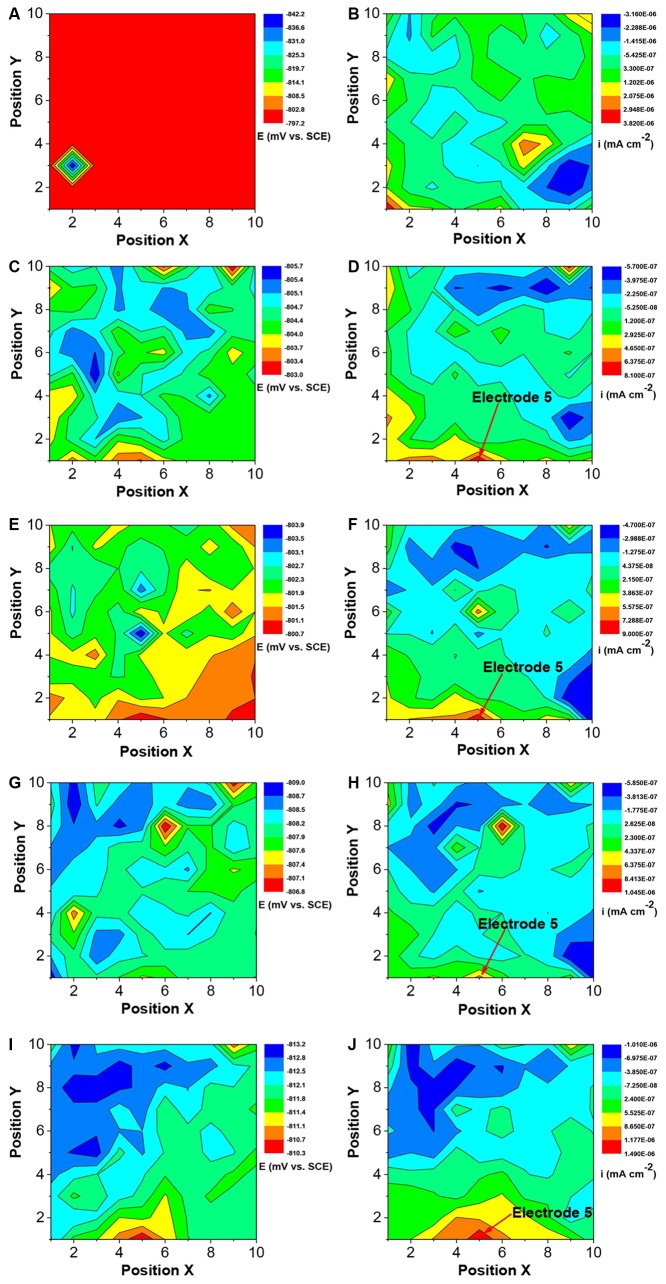
Changes of corrosion potential **(A,C,E,G,I)** and galvanic current **(B,D,F,H,J)** distributions of Q235 WBE with incubation time in the absence of SRB in CO_2_-saturated simulated formation water: **(A,B)** 1 day; **(C,D)** 4 days; **(E,F)** 7 days; **(G,H)** 10 days, and **(E,F)** 14 days.

The changes in the corrosion potential and galvanic current distribution maps of Q235 WBE in the absence of SRB are shown in Figure 12. It is seen that in the first day of incubation, the potentials were between −842 mV vs. SCE and −787 mV vs. SCE (Figure 12A). Electrode 32 (center of the diamond in Figure 12A) had the most negative potential. The potential differences among all the electrodes were the biggest in Figure 12A compared those for other days. In the galvanic current distribution maps (Figure 12B), it is seen that the total anodic area (with positive current reading) was bigger than the total cathodic area, with the biggest anodic current density of 3.82 × 10^−6^ mA cm^−2^. After 4 days of incubation, the range of potentials remained in a narrow range surrounding −800 mV (Figure 12C,E,G,I). Furthermore, it is hard to recognize the anodic area from the potential distribution maps after 4 days of inoculation. However, the galvanic current distribution maps indicate that the total anodic area was always bigger than the total cathodic area and most anodic areas shifted with time (Figure 12D,F,G,J). There were exceptions. For example, Electrode 5 corresponded to an obviously anodic dissolution site after 4 days of incubation, indicating localized corrosion. After 14 days of incubation, the anodic current density of electrode 5 is 1.49 × 10^−6^ mA cm^−2^. These results indicate that both general corrosion and localized corrosion occurred in UDC in the absence of SRB.

**FIGURE 13:**
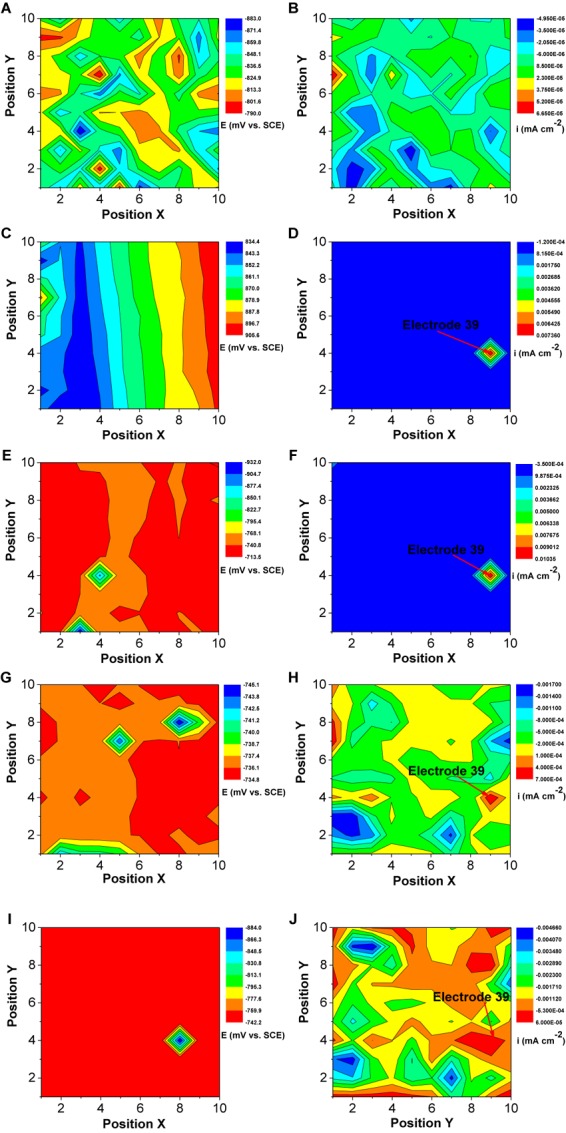
Changes of corrosion potential **(A,C,E,G,I)** and galvanic current **(B,D,F,H,J)** distributions of Q235 WBE with incubation time in the presence of SRB in CO_2_-saturated simulations formation water: **(A,B)** 1 day, **(C,D)** 4 days, **(E,F)** 7 days, **(G,H)** 10 days, and **(E,F)** 14 days.

Figure 13 shows the changes of corrosion potential and galvanic current distribution of Q235 WBE with incubation time in the presence of SRB. It is seen that in the first day of incubation, the potentials were between the range of 883 mV vs. SCE and 790 mV vs. SCE (Figure 13A), which is bigger than those for other incubation days. In the galvanic current distribution maps, it is seen that the total anodic area was bigger than the total cathodic area (Figure 13B). The maximum anodic current density of 6.65 × 10^−5^ mA cm^−2^ (Figure 13B) in the first day in the presence of SRB was much bigger than that for the control (Figure 12B), which indicated that SRB have contributed considerably to corrosion in the initial 1 day. With the increase of incubation time, the potential range in the presence of SRB (Figure 13C,E,G,I) was the biggest on day 7. With the increase of incubation time, the total anodic area decreased quickly. The maximum anodic dissolution current density site occurred at electrode 39 with a maximum value of 1.0 × 10^−6^ mA cm^−2^ on day 7 (Figure 13F). It was four orders of magnitude higher than that of the control (Figure 12F). This indicates that SRB significantly promoted localized UDC. The maximum anodic current density decreased after 7 days of incubation (Figure 13H,J), and the anodic areas also changed with time.

## Discussion

### Corrosion Behavior in the Absence of SRB

The weight loss result (Figure 4) indicated a light UDC of the control specimen. The SEM image (Figure 2a) of the corrosion product film and EIS analysis results (Figure 8A,B) indicated the formation of a protective film. Such a film can inhibit further corrosion (Liu et al., 2016a). XRD results (Figure 5A) indicated that the corrosion products were mainly composed of CaCO_3_, FePO_4_, FeOOH with a little FeCO_3_. FePO_4_ and FeOOH could be the result of oxidation of (Fe)_3_(PO_4_)_2_ and Fe(OH)_2_ during the specimen preparation process before the XRD analysis. In the anaerobic CO_2_-saturated simulated formation water, the anodic and cathodic reactions of carbon steel corrosion can be described below (Nešić, 2007; Tang et al., 2017):

Anodic reaction:

(2) $$\text{Fe}\to {\text{Fe}}^{2+}+2{\text{e}}^{-}$$

Cathodic reactions:

(3) $$2{\text{H}}_{2}\text{O}+2{\text{e}}^{-}\to 2{\text{OH}}^{-}+{\text{H}}_{2}$$

(4) $$2{\text{H}}_{2}{\text{CO}}_{3}+2{\text{e}}^{-}\to {\text{H}}_{2}+2{\text{HCO}}_{3}{}^{-}$$

(5) $$2{\text{HCO}}_{3}{}^{-}+2{\text{e}}^{-}\to 2{\text{CO}}_{3}{}^{2-}+{\text{H}}_{2}$$

CaCO_3_, (Fe)_3_(PO_4_)_2_ and Fe(OH)_2_ could be attributed to the following reactions:

(6) $${\text{Ca}}^{2+}+\text{CO}3^{2-}\to {\text{CaCO}}_{3}$$

(7) $$3{\text{Fe}}^{2+}+2{\text{PO}}_{4}{}^{3-}\to {\left(\text{Fe}\right)}_{3}{\left({\text{PO}}_{4}\right)}_{2}$$

(8) $${\text{Fe}}^{2+}+2{\text{OH}}^{-}\to \text{Fe}{\left(\text{OH}\right)}_{2}$$

Once a dense CaCO_3_ film forms on a steel surface, the iron dissolution rate slows down. Galvanic current distribution maps of WBE (Figure 12) showed lower anodic current densities compared with those in the presence of SRB that resulted in the disappearance of the dense corrosion product film. Usually, FeCO_3_ film passivates a carbon steel surface in CO_2_ corrosion. But in this case, there was not much corrosion in the absence of SRB, so there was not much FeCO_3_ in the corrosion product film. The larger total anodic area compared to the total cathodic care shown in Figure 12 indicates general corrosion. Furthermore, some local areas (e.g., electrode 5) had a much higher anodic current density, suggesting to localized corrosion, which was observed from the corrosion morphology in Figure 6. Localized corrosion is often found in UDC (Vera et al., 2012; Pandarinathan et al., 2013b). Han et al. (2013) found that the pitting corrosion under sand coming from the galvanic effect. In this paper, the galvanic effect was detected by WBE. Clay also can influence the corrosion process because it is involved in the mass transfer of corrosion products (Jeannin et al., 2010).

### Corrosion Behavior in the Presence of SRB

Many previous reports have verified that SRB can considerably accelerate steel corrosion, especially localized corrosion (Enning and Garrelfs, 2014; Li H. et al., 2016). In this work, both the general corrosion and localized corrosion were considerably enhanced by SRB based on weight loss (Figure 4) and corrosion morphology (Figure 6a,b). These indicated that SRB promoted UDC. SRB corrosion under deposit is different from that without deposit. Deposit on a steel surface is a mass transfer barrier for nutrients such as organic carbon (Usher et al., 2014), because of the tortuosity of the deposit layer (Batmanghelich et al., 2017). This means that the sessile SRB cells under deposit only can get limited nutrients. Some previous reports have demonstrated that SRB are more corrosive when they are starved of organic carbons because they switch to Fe^0^ as an energy source instead (Xu and Gu, 2014; Beese-Vasbender et al., 2015; Chen et al., 2015). This could be a key reason that the serious pitting corrosion was observed in the presence of SRB in this work (Figure 6a).

SRB corrosion is an electrochemical process (Liu et al., 2015a). Potentiodynamic polarization curves (Figure 11) indicated that SRB promoted both anodic and cathodic reactions, thus considerably accelerating corrosion. The anodic reaction is the dissolve of Fe^0^, and the cathodic reaction is reduction of sulfate as shown below (Zhang et al., 2015):

Cathodic reaction:

(9) $${\text{SO}}_{4}{}^{2-}+9{\text{H}}^{+}+8{\text{e}}^{-}\to {\text{HS}}^{-}+4{\text{H}}_{2}\text{O}$$

This means that the reduction rate of sulfate catalyzed by SRB contributed to the overall cathodic reaction process. Potential and galvanic current distribution maps of WBE (Figure 13) indicated that galvanic corrosion under deposit in the presence of SRB promoted the localized corrosion. The maximum anodic current density appeared on day 7, and then decreased with time gradually (Figure 13). The growth curve of SRB in a previous report indicated (Liu et al., 2015b) that SRB cell mass reached a maximum value after 7 days of incubation, then it started to decline. This suggests that the galvanic corrosion under deposit was related to the SRB activity.

Extracellular electron transfer for SRB corrosion has been verified by some previous reports (Deng et al., 2015; Li et al., 2015). This means that SRB can also indirectly obtain electrons from Fe^0^, thus enhancing steel corrosion. FeS, the typical corrosion products of SRB, also can transfer electrons duo its higher electroconductibility. The distribution of sessile SRB cells is heterogeneous (Dong et al., 2011b), which means that some SRB cells can directly attach on steel surface while some in biofilm are away from steel surface. The results of potential and galvanic current distribution maps of WBE (Figure 12, 13) indicated that SRB considerably accelerated the galvanic corrosion. It is very difficult for sessile SRB cells attached on steel surface capture sulfate due to the hindering of biofilm and deposit. So, the electrons directly obtained from Fe^0^ by SRB can transfer to the other SRB which can capture sulfate more easily through extracellular electron transfer. These means that the sessile SRB cells with different locations in biofilm have a synergistic effect accelerating galvanic corrosion. And SRB activity will decrease with the increase of incubation time, thus causing the decrease of galvanic current density (Figure 13).

## Conclusion

Both the general corrosion and localized corrosion of the control specimen under deposit were light in the absence of SRB. From the weight loss results, steel corrosion in the presence of SRB was approximately 6 times of that for the control. The maximum pit depth in the presence of SRB was approximately 7.7 times of that for the control. The formation of a dense corrosion product film on the control specimen surface was responsible for the light corrosion. In the presence of SRB, the corrosion products were mainly composed of FeS and Fe(OH)_2_. They did not form a dense protective film. Potentiodynamic polarization curves indicated that both the anodic and cathodic reactions were accelerated in the presence of SRB, thus accelerated corrosion. WBE measurements results indicated that the galvanic effect in the presence of SRB promoted the localized corrosion under deposit. After 7 days of incubation, the anodic current density decreased gradually, which coincided with the declined of sessile SRB activity.

## Data Availability

The raw data supporting the conclusions of this manuscript will be made available by the authors, without undue reservation, to any qualified researcher.

## Author Contributions

HwL, HfL, and GM designed the experiments. HwL carried out the experiments and finished the manuscript writing. HwL, GM, and WL analyzed the experimental data. HfL and TG helped to revise the manuscript and gave much help in the discussion part.

## Conflict of Interest Statement

The authors declare that the research was conducted in the absence of any commercial or financial relationships that could be construed as a potential conflict of interest.
