# Corrigendum: Microbiologically Influenced Corrosion of Carbon Steel Beneath a Deposit in CO_2_-saturated Formation Water Containing *Desulfotomaculum nigrificans*

**DOI:** 10.3389/fmicb.2019.01653

**Published:** 2019-07-17

**Authors:** Hongwei Liu, Guozhuo Meng, Weihua Li, Tingyue Gu, Hongfang Liu

**Affiliations:** ^1^School of Chemical Engineering and Technology, Sun Yat-sen University, Zhuhai, China; ^2^Department of Chemical and Biomolecular Engineering, Institute for Corrosion and Multiphase Technology, Ohio University, Athens, OH, United States; ^3^Key Laboratory of Material Chemistry for Energy Conversion and Storage, Ministry of Education, Hubei Key Laboratory of Materials Chemistry and Service Failure, School of Chemistry and Chemical Engineering, Huazhong University of Science & Technology, Wuhan, China

**Keywords:** sulfate reducing bacteria, biofilm, carbon steel, under deposit corrosion, microbiological corrosion

In the original article, there was a mistake in Figure 12 as published. Figures 12A–H are the same as Figures 13A–H. The correct Figure 12 appears below.

**Figure 1:**
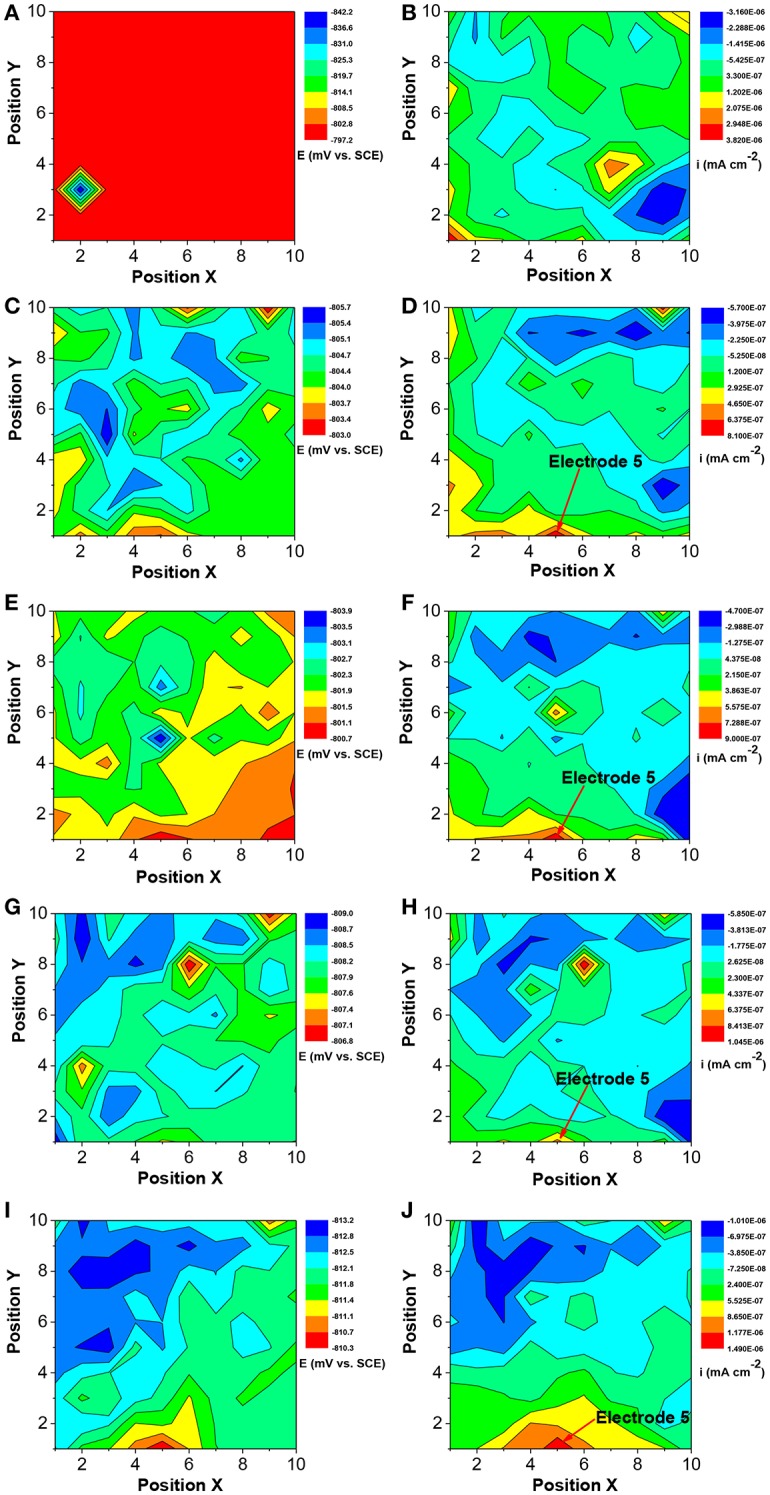
Changes of corrosion potential **(A,C,E,G,I)** and galvanic current **(B,D,F,H,J)** distributions of Q235 WBE with incubation time in the absence of SRB in CO_2_-saturated simulated formation water: **(A,B)** 1 day; **(C,D)** 4 days; **(E,F)** 7 days; **(G,H)** 10 days and **(E,F)** 14 days.

The authors apologize for this error and state that this does not change the scientific conclusions of the article in any way. The original article has been updated.

